# Non-destructive visualization of internal structural changes in humidified magnesium oxide tablets using X-ray computed tomography

**DOI:** 10.1038/s41598-024-56949-8

**Published:** 2024-03-15

**Authors:** Takahiro Amemiya, Kazuhiro Suzuki, Takashi Tomita

**Affiliations:** 1 Department of Pharmacy, The University of Tokyo Hospital, Tokyo, Japan; 2 Semiconductor Evaluation Laboratory, Evaluation and Analysis Technology Center, Toshiba Nanoanalysis Corporation, Kanagawa, Japan; 3 Department of Pharmacy, International University of Health and Welfare Mita Hospital, Tokyo, Japan; 4 Department of Pharmaceutical Sciences, School of Pharmacy, International University of Health and Welfare, Tochigi, Japan

**Keywords:** Medical research, Materials science

## Abstract

Detailed examinations of the internal structure of tablets are imperative for comprehending their formulation, physical attributes, and ensuring their safe utilization. While X-ray computed tomography (CT) is valuable for noninvasively analyzing internal structural changes, the influence of humidity on these structural changes remains unexplored. Accordingly, we aimed to assess the viability of X-ray CT in non-destructively evaluating the internal structure of humidified magnesium oxide (MgO) tablets. MgO tablets were subjected to conditions of 40 °C and 75% humidity for 7 days, weighed pre- and post-humidification, and subsequently stored at room temperature (22–27 °C) until day 90. Their internal structure was evaluated using X-ray CT. We observed a substantial increase in the weight of MgO tablets concomitant with moisture absorption, with minimal changes observed upon storage at room temperature. The skewness reduced immediately post-moisture absorption, remained almost the same post-storage at room temperature, and failed to revert to pre-humidification levels during the storage period. These findings highlight the utility of X-ray CT as an effective tool for non-destructive, three-dimensional, and detailed evaluation of internal structural transformations in MgO tablets.

## Introduction

The oral administration route is widely preferred owing to its convenience in both administration and manufacturing, with tablets being the favored solid dosage form^1^. The advantages of tablets include the ability to control the site and time of drug release, based on factors such as solubility and sustained release. Additionally, tablets provide the ability to mask taste^2^. However, tablets are susceptible to environmental factors, specifically temperature and humidity, which can impact their physical properties, including reduced hardness and altered disintegration time^3–5^.

Magnesium oxide (MgO) tablets, widely recognized for their antacid and laxative effects, significantly improve the quality of life for patients suffering from constipation, with an estimated annual treatment of 10 million patients in Japan^6^. MgO tablets are particularly hygroscopic and increase in hardness and weight under the influence of humidity^7^. Consequently, humidity-induced difficulties in disintegration may pose challenges for patients with impaired swallowing function.

In addition to active pharmaceutical ingredients, tablets typically incorporate various excipients depending on their intended use^8^. Therefore, elucidating the internal structure of a drug product is crucial for comprehending its formulation, function, and physical properties. Despite this importance, there is currently no established method for observing the detailed internal structure of tablets.

Recent studies have explored the utility of X-ray computed tomography (CT) as a noninvasive method for analyzing the gradual internal structural changes in tablets^9^. Using the penetrating power of X-rays, X-ray radiography helps acquire two-dimensional transmission images of the internal structure of the sample from various angles. The sequential imaging data are then used to non-destructively construct a three-dimensional (3D) X-ray CT image, with high-luminance X-ray sources such as synchrotron radiation enabling rapid measurements and accurate capture of temporal changes^10,11^. However, we lack studies evaluating the humidification-induced internal structural changes in tablets. Therefore, we aimed to determine the impact of humidity on the internal structure of tablets, particularly MgO tablets, using X-ray CT. We believe that our findings may have broader implications for pharmaceutical formulation, quality control, and patient safety.

## Results

### Weight change of MgO under humidified conditions

**Figure 1:**
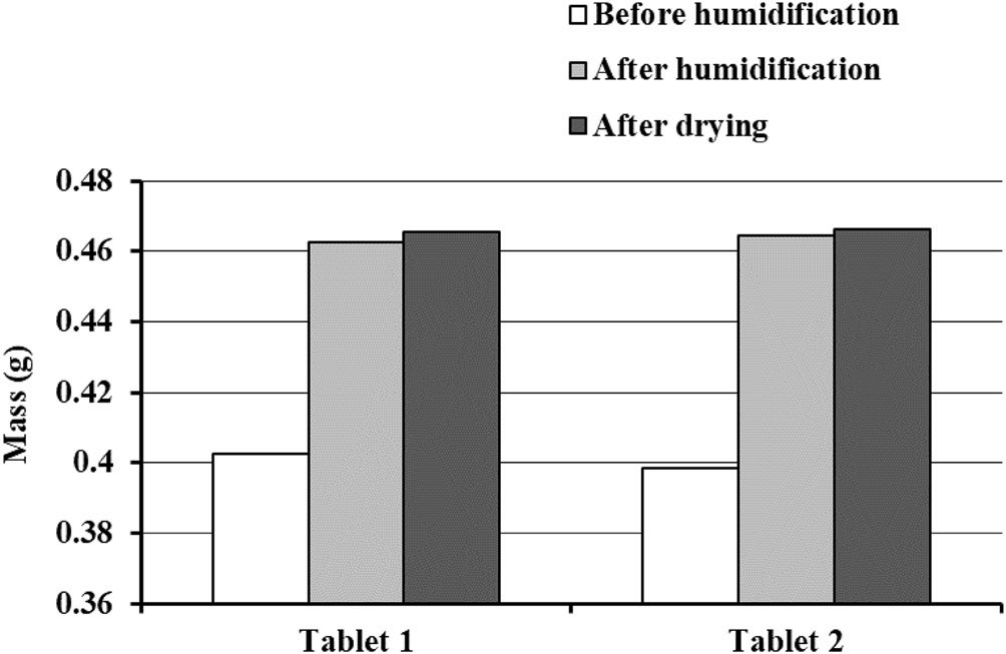
Weight changes of MgO tablets before and after humidification and after drying. The mass of the MgO tablets was measured before humidification (day 0), immediately after humidification (day 7), and on day 90 after storage at room temperature. Humidification was performed at 40 °C and 75% relative humidity for 7 days.

The mass of MgO tablets 1 and 2 increased after storage for 7 days under humid conditions (40 ℃ and 75% relative humidity; Fig. 1, data in tablets 1 and 2 illustrate that humidity caused an average 1.157-fold increase in weight. Subsequently, the tablets that absorbed humidity were dried at room temperature, resulting in minimal change in weight (Fig. 1).

### Comparison of X-ray CT images of MgO tablets before and after moisture absorption experiment

**Figure 2:**
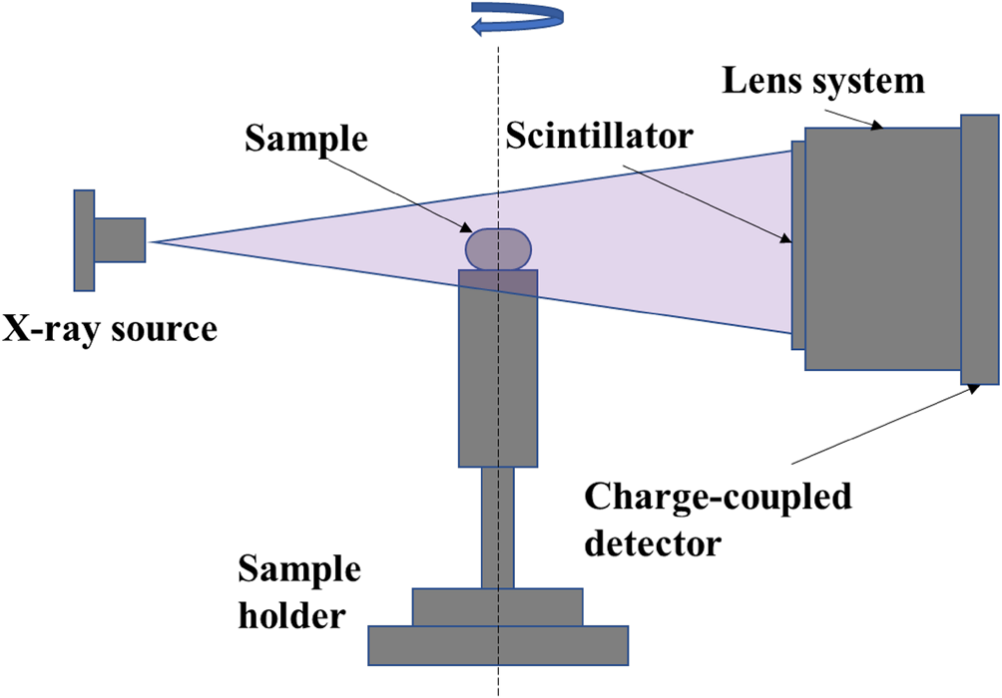
Diagram of the 3D X-ray microscope. The X-ray source emits a cone beam of X-rays, and the sample absorbs a portion of these X-rays. Subsequently, the scintillator transforms X-rays into light, and a fraction of this light passes through the lens system to reach the charge-coupled detector, thereby generating X-ray radiographs. The sample holder is capable of a 360° rotation, facilitating comprehensive observation of the sample from all directions.

**Figure 3:**
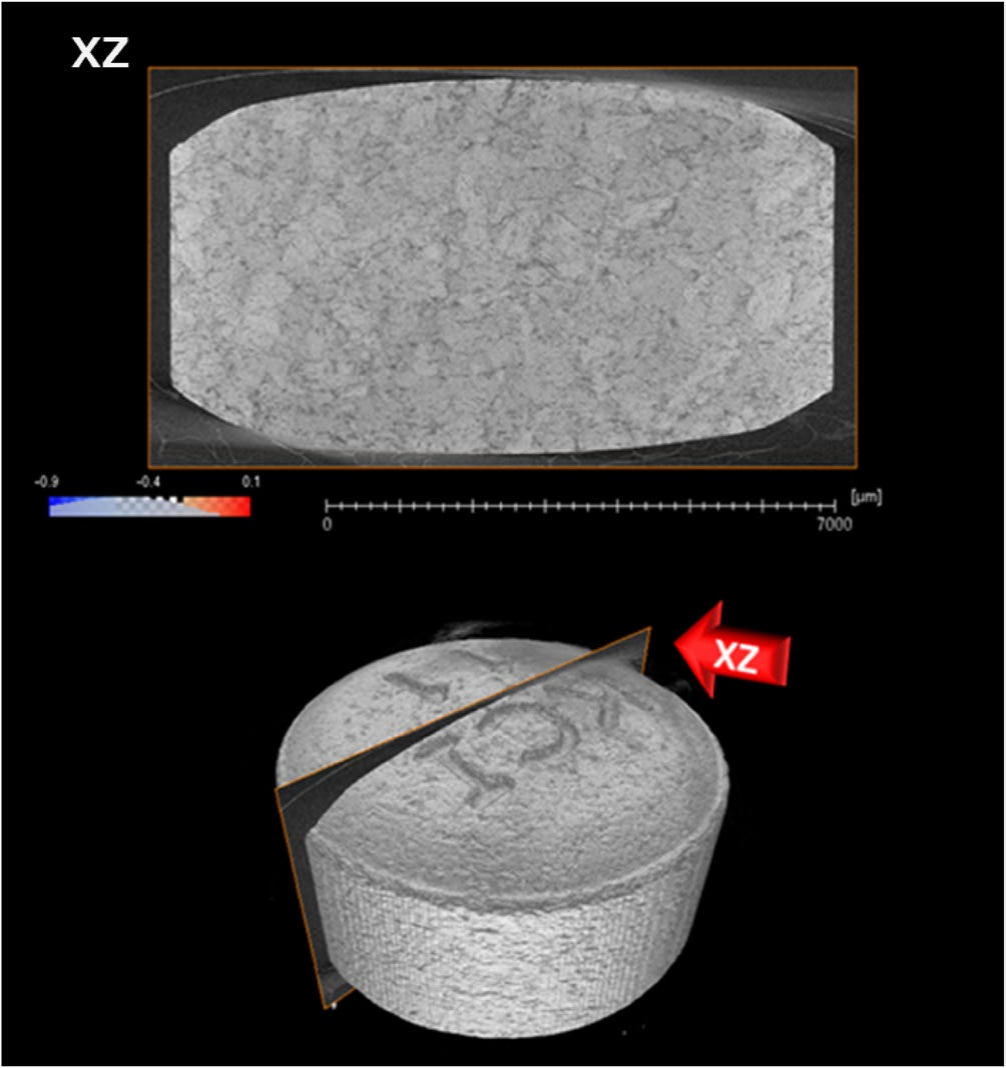
X-ray CT data of the MgO tablet. X-ray radiography of the sample was performed using a ZEISS Xradia 520 Versa (ZEISS) with a voltage of 60 kV and a power of 5.0 W. X-ray CT data were computed from the obtained X-ray radiographs. The virtual slice and a three-dimensional representation are presented.

**Figure 4:**
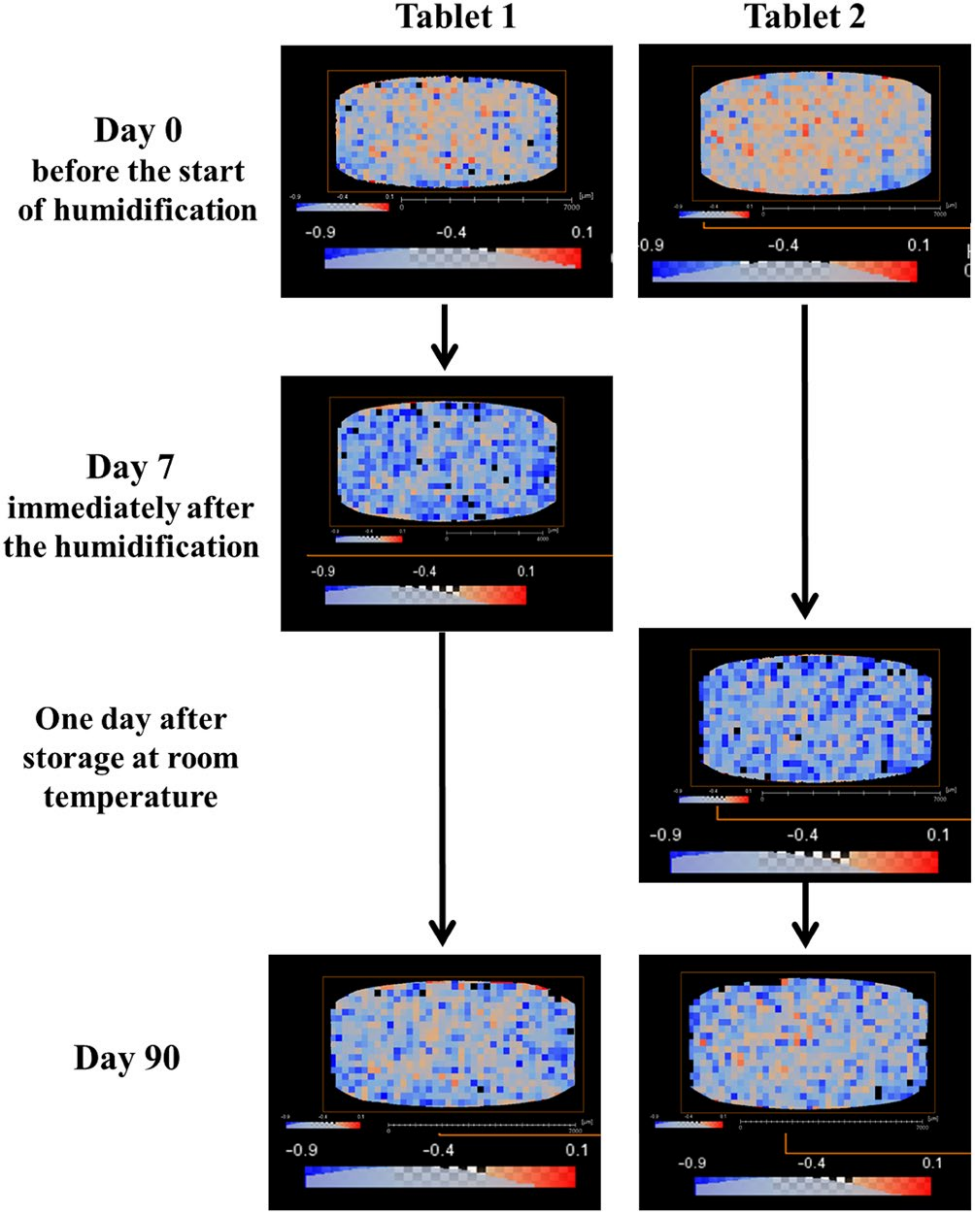
X-ray CT images and skewness distribution of MgO tablets before and after humidification and after drying. X-ray radiography of the sample was performed on a cross-section of the MgO tablets at a voltage of 60 kV and a power of 5.0 W using a ZEISS Xradia 520 Versa (ZEISS). X-ray CT data were computed from X-ray radiographs.

**Figure 5:**
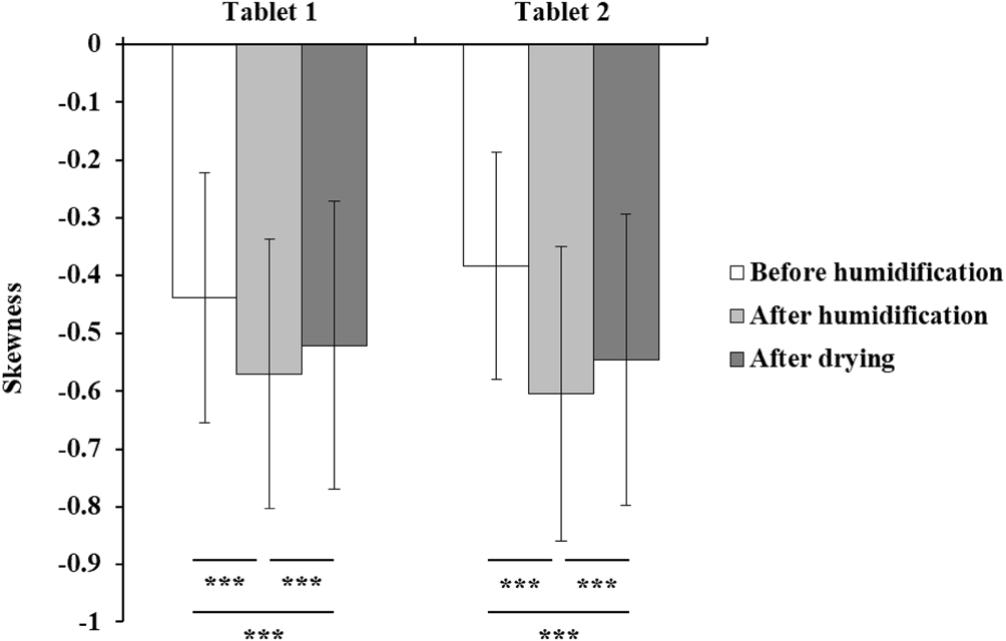
Average value of skewness in MgO tablets before and after humidification and after drying. The skewness of the tablets was calculated based on the X-ray absorptivity (brightness) of each cube from the X-ray CT data. Data are expressed as the mean ± S.D. ***p < 0.001 represents a significant difference. The skewness of tablet 2 after the moisture absorption experiment was measured one day after the end of the moisture absorption experiment.

The 3D X-ray microscopic configuration used in this study is illustrated in Fig. 2. Voxel data of the tablets were segmented into multiple cubes, and skewness was calculated based on the X-ray absorption coefficients within each cube (Fig. 3). The time series of skewness for tablets 1 and 2 before and after humidification, and after subsequent drying, was calculated using X-ray radiography (Fig. 4). As the validation analyses concerning various voxel cube dimensions exhibited an identical trend, the X-ray CT data were segregated into multiple 40 × 40 × 40 voxel cubes in this study (Supplementary Fig. 1). Tablets 1 and 2 exhibited similar skewness prior to moisture absorption (Figs. 4, 5). After the 7-day moisture absorption experiment, tablet 1 displayed a decrease in skewness compared with that during its pre-absorption state (Figs. 4, 5). Similarly, tablet 2, observed one day after the experiment, exhibited reduced skewness compared with that during its pre-absorption state, similar to the pattern observed in tablet 1 immediately after the experiment (Figs. 4, 5). Finally, the reduced skewness did not revert to the pre-moisture absorption levels after drying at room temperature (Figs. 4, 5).

### Evaluation of histogram based on depth-specific X-ray CT images of MgO tablets before and after moisture absorption experiment

**Figure 6:**
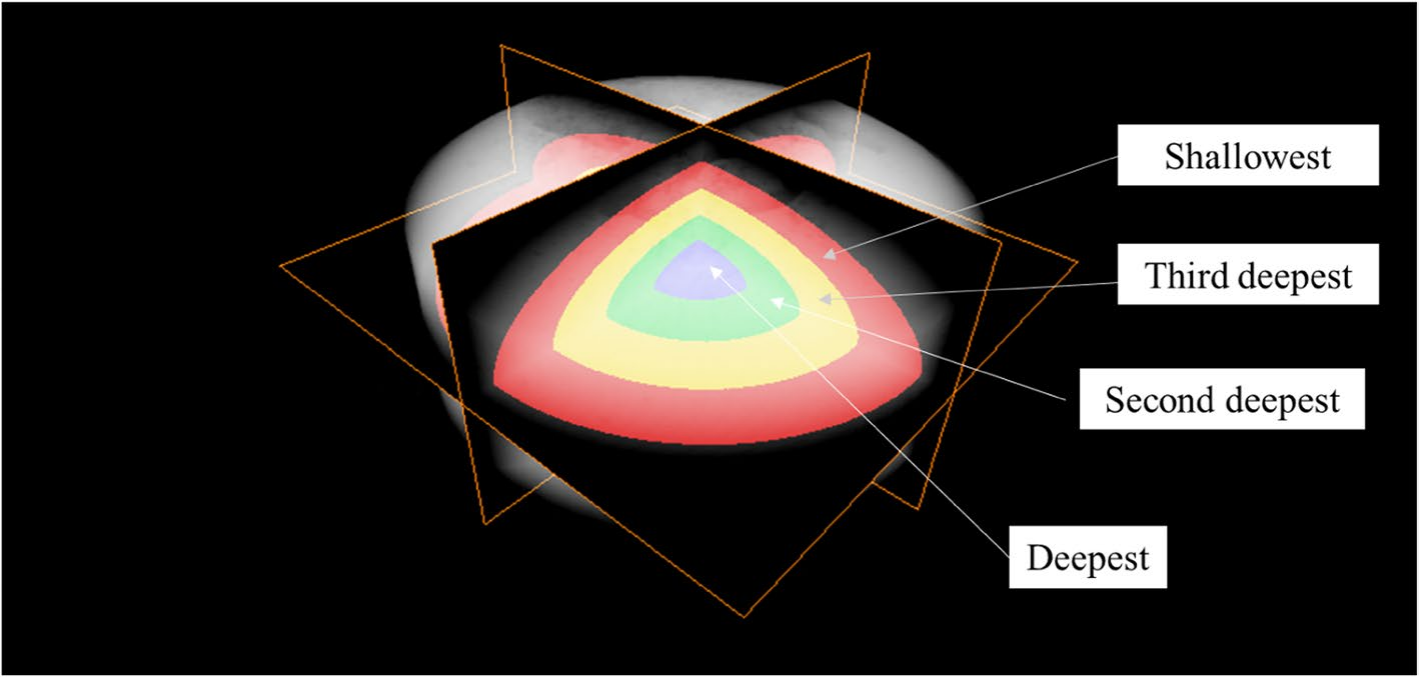
Cross-sectional view of the four laminae of the MgO tablet. The illustration depicts a low-opacity, three-dimensional view of the tablet and four slices of its sections.

**Figure 7:**
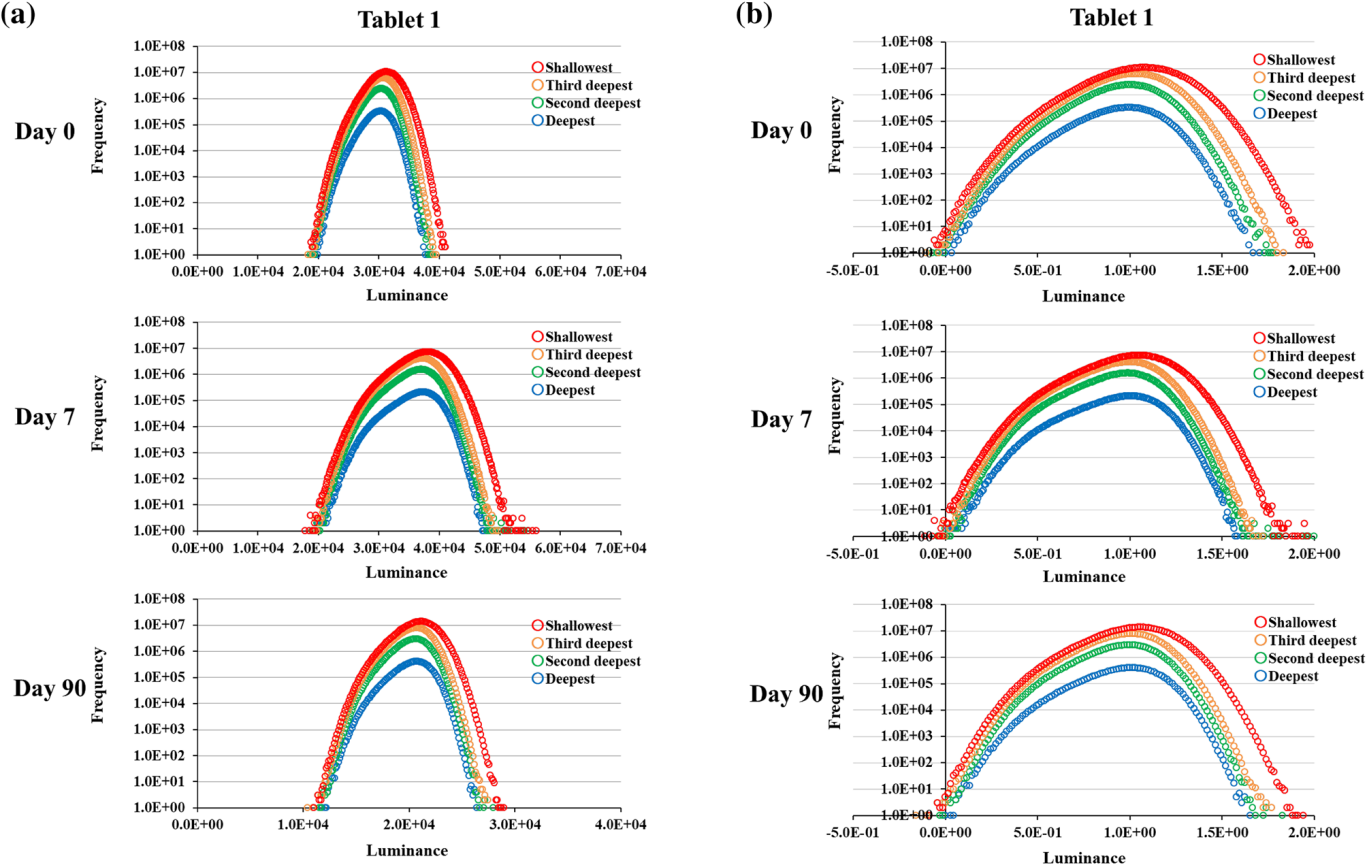

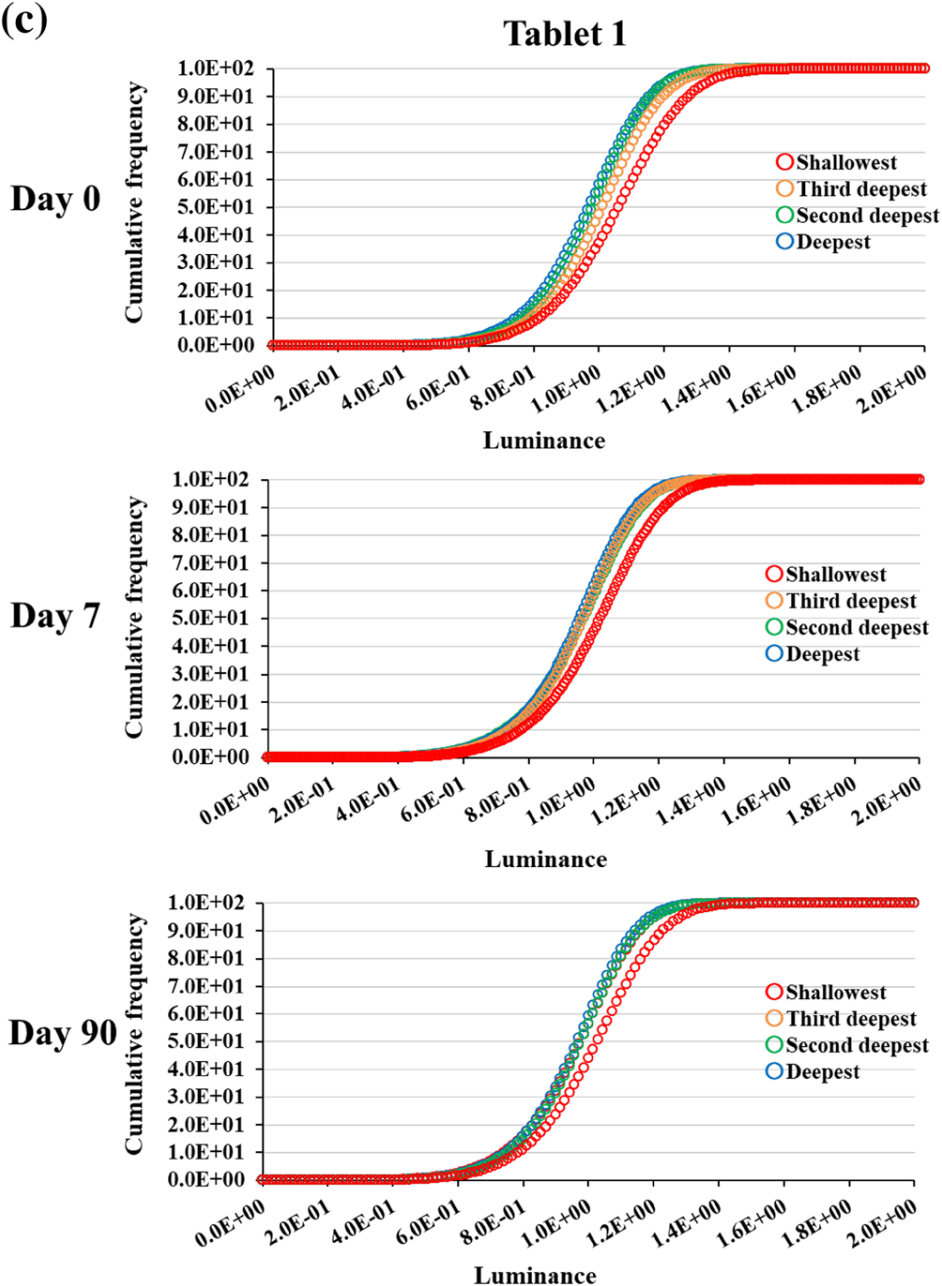
Depth-specific histograms based on X-ray CT data of the MgO tablet before and after humidification and after drying. (**a**) Histograms whose luminance remains unconverted. Panel (**a**) displays the unprocessed data. The luminance is represented by 16-bit data. (**b**) Histograms whose luminance is converted for their comparison. To facilitate the comparison of the data from days 0, 7, and 90, normalizing the luminance was necessary. The standardized data is depicted in (**b**). (**c**) Cumulative frequency distributions of the histograms presented in (**b**). Panel (**c**) was created to portray the magnitude of the high-luminance volume. The large inner part of the tablet in the X-ray CT data, consisting of voxels, was divided into the deepest, second deepest, third deepest, and shallowest portions. Each voxel had a luminance. Histograms depicting the luminance distributions in the portions were created.

A low-opacity, three-dimensional view of the tablet and four segments of its laminae were visualized (Fig. 6). Histograms were constructed using X-ray CT absorbance and frequency at various depths of the MgO tablets before and after moisture absorption and post-drying (Fig. 7a). Consistently, the X-ray absorption rate was higher on the exterior of the tablets and lower on the interior in all measurements. No discernible changes were observed in the histograms concerning the depth of the MgO tablets before and after moisture absorption and after drying. Prior to moisture absorption, the histogram displayed a normal distribution; however, on day 7, immediately after the moisture absorption experiment, the X-ray absorption rate decreased, and the peak value shifted to the right. Furthermore, this shift persisted on day 90, indicating that it did not revert to pre-humidity absorption levels after drying at room temperature.

When plotting the histograms, a linear transformation of luminance was applied for enhanced clarity, as demonstrated in Fig. 7b. This transformation ensured that the minimum and most frequent luminance in the histogram from the deepest part of the tablet on each date were zero and one, respectively. The cumulative frequency graphs of these histograms are illustrated in Fig. 7c. The aforementioned results are consistent with those documented in Tablet 2 (Supplementary Fig. 2).

## Discussion

X-ray CT has been previously demonstrated as an effective noninvasive tool for analyzing internal structural changes in tablets over time; however, its applicability to assess structural changes under humidification remained uncertain. In this study, we demonstrated the utility of X-ray CT for non-destructively observing detailed internal structural changes in moisture-absorbed MgO tablets. Moisture absorption resulted in a notable increase in the weight of MgO tablets, potentially attributed to the formation of hydromagnesite^7^. The change in mass under hygroscopic conditions is attributed to the absorption of water and carbon dioxide gas in MgO, leading to the preferential formation of hydromagnesite on the tablet surface during early storage^4^. Moreover, hydromagnesite formation was observed on the tablet surfaces, as well as at depths of 0.4 or 0.8 mm^4^, indicating its potential involvement in the weight increase after moisture absorption experiments.

The 3D X-ray CT image of the MgO tablet, composed of small voxels with brightness approximately proportional to their X-ray absorption, allowed the presentation of overall characteristics through histograms. We used skewness as a parameter for quantification. Skewness, a normalized metric for quantifying the shape of histograms, offers two key advantages for analyzing X-ray CT datasets. First, it facilitates a straightforward comparison of skewness across different datasets. Second, skewness facilitates the density analysis of samples in X-ray CT data. In summary, large- and small-density areas contribute positively and negatively to the skewness of data, respectively. The skewness of these histograms provided insights into differences in tablet cross-section density, revealing a decrease after moisture absorption compared with that during pre-moisture absorption conditions (Figs. 4, 5). Furthermore, a decrease in X-ray absorptivity at all depths owing to moisture absorption was evident in the histogram of X-ray absorptivity (Fig. 7), notwithstanding the consideration of beam-hardening artifacts in CT^12^. Cumulative frequency distributions in Fig. 7c demonstrated a higher luminance at a cumulative frequency of 90% from the shallowest part on day 0 than on days 7 or 90, indicating a persistent shift to the right. This shift aligns with the larger skewness on day 0, as depicted in Fig. 5, suggesting that the transformed histograms are consistent with the study results. Given the permeability of MgO tablets to water, the early formation of hydromagnesite during moisture absorption^7^, and the lower true density of hydromagnesite (2.2 g/mL) compared with that of MgO (3.17 g/mL)^4^, the moisture-absorbed MgO tablets may likely exhibit lower density, leading to the observed decrease in skewness. Conversely, the precise determinant underlying the post-drying modification in histogram and skewness remains unknown (Fig. 5). Nevertheless, pharmaceutical additives may have effectuated an elevation in skewness upon drying^13^, thereby potentially affecting the X-ray CT measurements in the present study.

The study results indicated minimal weight change in the MgO tablets after moisture absorption, even after drying (Fig. 1). MgO exhibits a rapid weight increase with higher relative humidity; however, the slight decrease in weight with decreasing relative humidity is likely attributed to the formation of hydromagnesite, increasing tablet hardness and decreasing tablet porosity through strong cross-links between powder particles^4^. The formed hydromagnesite likely impedes water dislodgment, contributing to the limited weight change observed during drying conditions in this study.

X-ray CT, similar to magnetic resonance imaging (MRI), has emerged as an advanced non-destructive testing technology^14^. Currently, CT with excellent resolution is actively used for the comprehensive analysis of the human body and agricultural crops, facilitated by advancements in X-ray tubes and detectors^14^. In MRI, the magnetization of hydrogen atoms in a sample is achieved through a robust magnetic field, generating MR images based on signal intensity variations corresponding to the strength of magnetization^15^. MRI is recognized as a commonly utilized imaging approach in clinical practice to identify diverse abnormalities in the body and is characterized by sharp image contrast^16^. However, the observed variability in image quality with changes in water content is a notable limitation^17^.

Additionally, the necessity for an accessory coil outside the sample to obtain a uniform magnetic field in MRI restricts the size and shape of the sample^14^. Hence, X-ray CT is considered superior to MRI for evaluating internal structural changes in tablets. X-ray CT applied to orally disintegrating (OD) tablets proves valuable for non-destructive assessment of liquid absorption and transport within these tablets^9^. In the future, X-ray CT may be used to evaluate the impact of moisture absorption on the dissolution of OD tablets. Moreover, the anticipated application of X-ray CT includes the evaluation of changes in the state of tablet coating layers and the particle size distribution within capsules. However, the limitations of this study may be analyzing the data at a single point in time, the lack of studies on tablets of different sizes, and other humidity conditions. Furthermore, the generalizability of the findings may be limited to MgO tablets, and extrapolating these results to other pharmaceutical formulations should be done cautiously.

In conclusion, our findings suggest that X-ray CT is a valuable non-destructive method for precisely evaluating the 3D internal structural changes in tablets. The X-ray CT analysis revealed that the decrease in the skewness of moisture-absorbed MgO tablets did not revert to their pre-absorption state, even after drying, highlighting its usefulness in the precise assessment of tablet internal structure. These findings provide valuable insights for pharmaceutical development, ensuring the efficacy and safety of MgO tablets, particularly when considering their applications in antacid and laxative effects. Additionally, the validation of X-ray CT as an effective tool for nondestructive and detailed evaluation of internal structural transformations opens avenues for improved quality control and formulation optimization in tablet manufacturing.

## Methods

### Materials

MgO tablets (330 mg) were procured from Magmitt Pharmaceutical Co., Ltd., Japan (lot.23A043).

### Humidification of the MgO tablet

Two MgO tablets, extracted from the press through packaging, were placed in cases and stored in an incubator (PL-3KT, ESPEC Corp., Osaka, Japan) at 40 °C and 75% relative humidity for 7 days. Subsequently, both tablets were transferred and stored at room temperature, and their masses were measured before and after humidification. The tablets were then stored at room temperature until day 90.

### X-ray CT measurement

The 3D X-ray microscope configuration is illustrated in Fig. 2. The MgO tablet was fixed to the sample holder and incrementally rotated until a full 360° angle was achieved. X-ray radiography was performed at each angle using a ZEISS Xradia 520 Versa X-ray microscope (Carl Zeiss X-ray Microscopy, Pleasanton, CA, USA) with a tube voltage of 60 kV and tube power of 5.0 W. The computed X-ray CT data had a voxel size of 6 × 6 × 6 μm^3^, where the brightness of each voxel corresponded to its X-ray absorption. The tablet portion extracted from the X-ray CT data was divided into multiple 40 × 40 × 40 voxel cubes, with cut surfaces representing the tablet surface. Different brightness distributions in each cube were expressed using brightness histograms. Skewness, a measure of histogram shape, was obtained for each cube. X-ray CT measurements were performed before humidification, immediately after humidification, one day after humidification, and after 90 days. An identical verification was conducted for the instances involving the 10 × 10 × 10 and 100 × 100 × 100 voxel cubes. Additional analyses involved dividing the large inner part of the tablet into four sections. The innermost section comprises a filled ellipsoid, whereas the others are hollow ellipsoids. Their centroids coincide with the center of the tablet. Avizo 2020.2 (Thermo Fisher Scientific, Waltham, MA, USA) was used for data analysis in this study.

### Statistical analysis

The data are presented as mean ± SD. Statistical analyses were performed using the Tukey–Kramer method in BellCurve for Excel, version 4.05 (Social Survey Research Information Co., Ltd., Japan). The threshold for statistical significance was established at *p* < 0.05.

### Supplementary Information

Supplementary Figures.

## Data Availability

The data that support the findings of this study are available from the corresponding author upon reasonable request.
