# Supplementary material for: Non-destructive visualization of internal structural changes in humidified magnesium oxide tablets using X-ray computed tomography
Source: Sci Rep. 2024 Mar 15;14:6339. doi: 10.1038/s41598-024-56949-8 (PMC10943080; doi:10.1038/s41598-024-56949-8)

Supplementary information for Non-destructive visualization of internal structural changes in humidified magnesium oxide tablets using X-ray computed tomography

Author: Takahiro Amemiya, Kazuhiro Suzuki, and Takashi Tomita*

*To whom correspondence should be addressed. E-mail: [ttomita@iuhw.ac.jp](mailto:ttomita@iuhw.ac.jp)

This file includes:

**Supplemental Figure 1.** Validation of X-ray CT images based on different binning sizes.

**Supplemental Figure 2.** Depth-specific histograms based on X-ray CT data of the MgO tablet before and after humidification and after drying.

**Supplemental Figure 1.** Validation of X-ray CT images based on different binning sizes.

1. X-ray CT images on Tablet 1 with voxel cubes of 10 x 10 x 10 and 100 x 100 x 100.


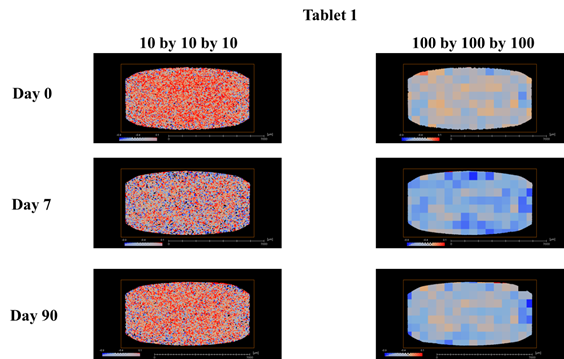


(b) X-ray CT images on Tablet 2 with voxel cubes of 10 x 10 x 10 and 100 x 100 x 100.
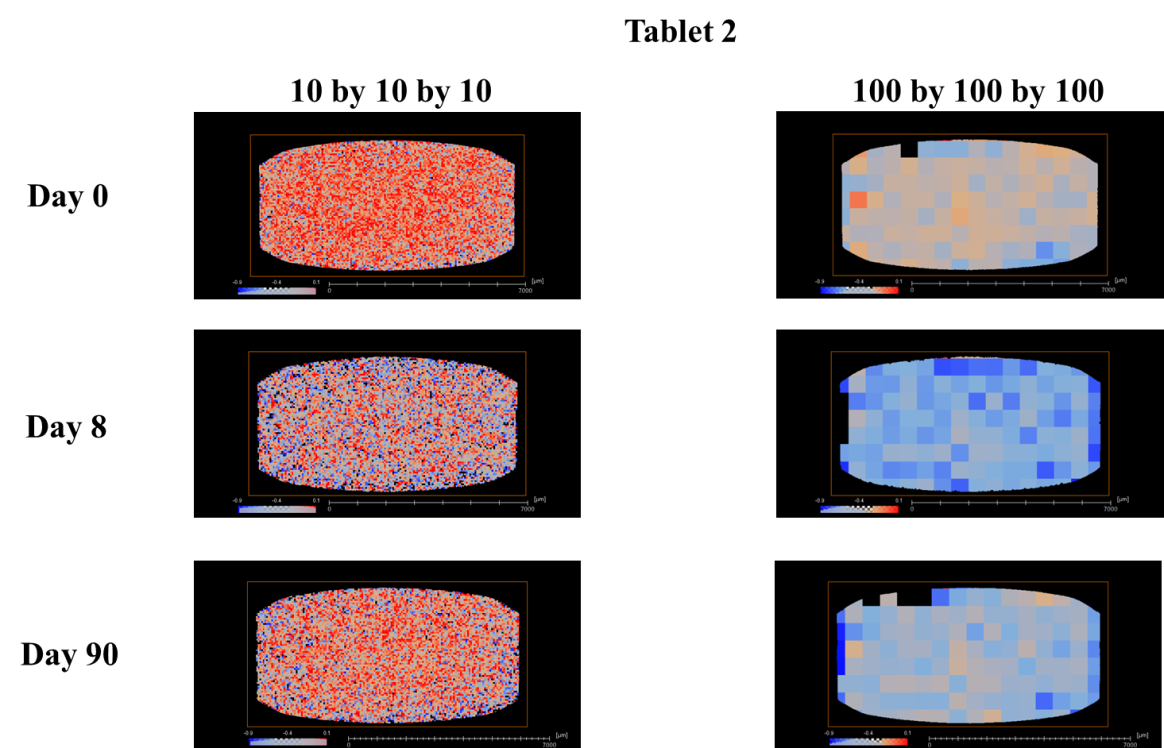


**Supplemental Figure 2.** Depth-specific histograms based on X-ray CT data of the MgO tablet before and after humidification and after drying.

(a) Histograms whose luminance remains unconverted.
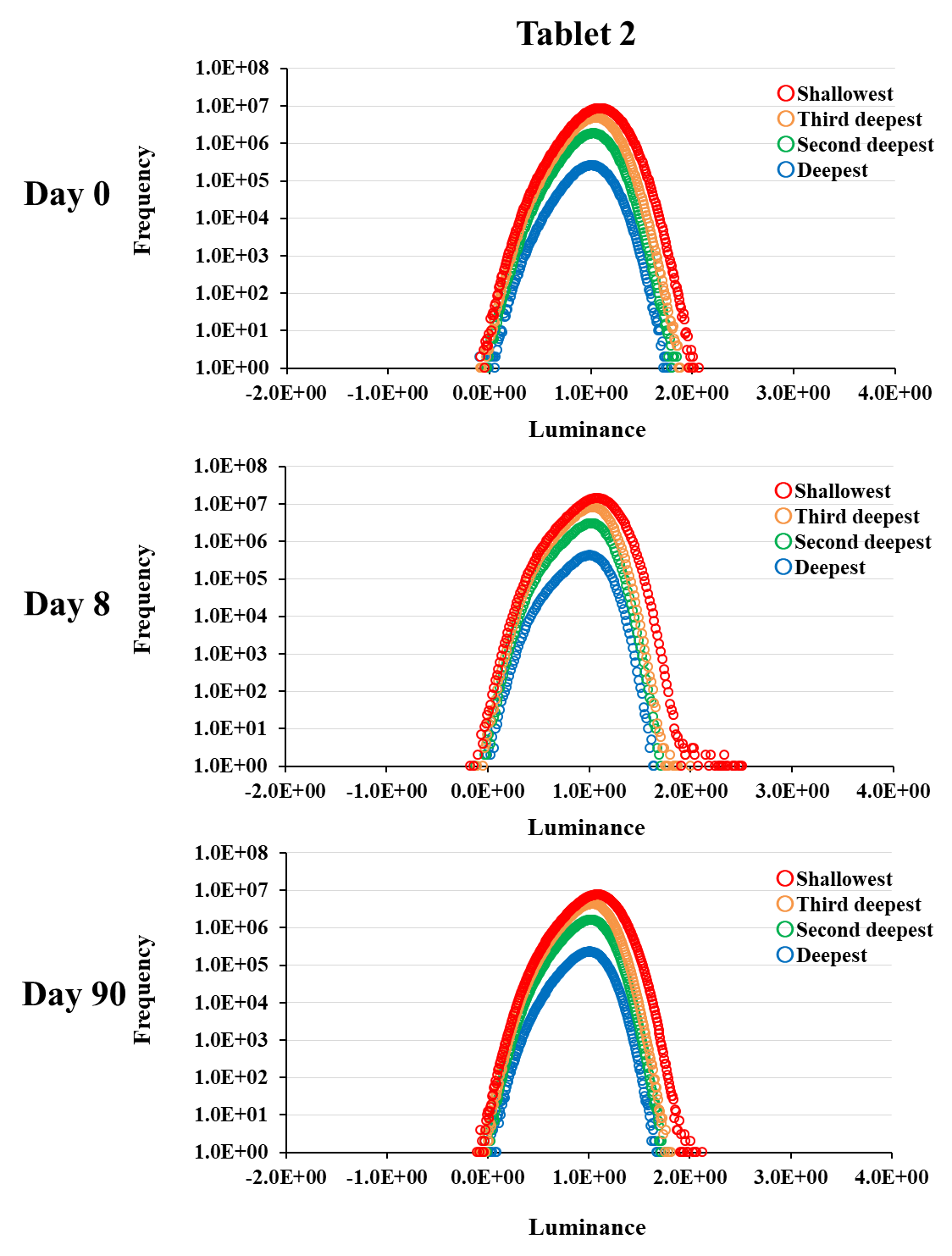


(b) Histograms whose luminance is converted for their comparison.


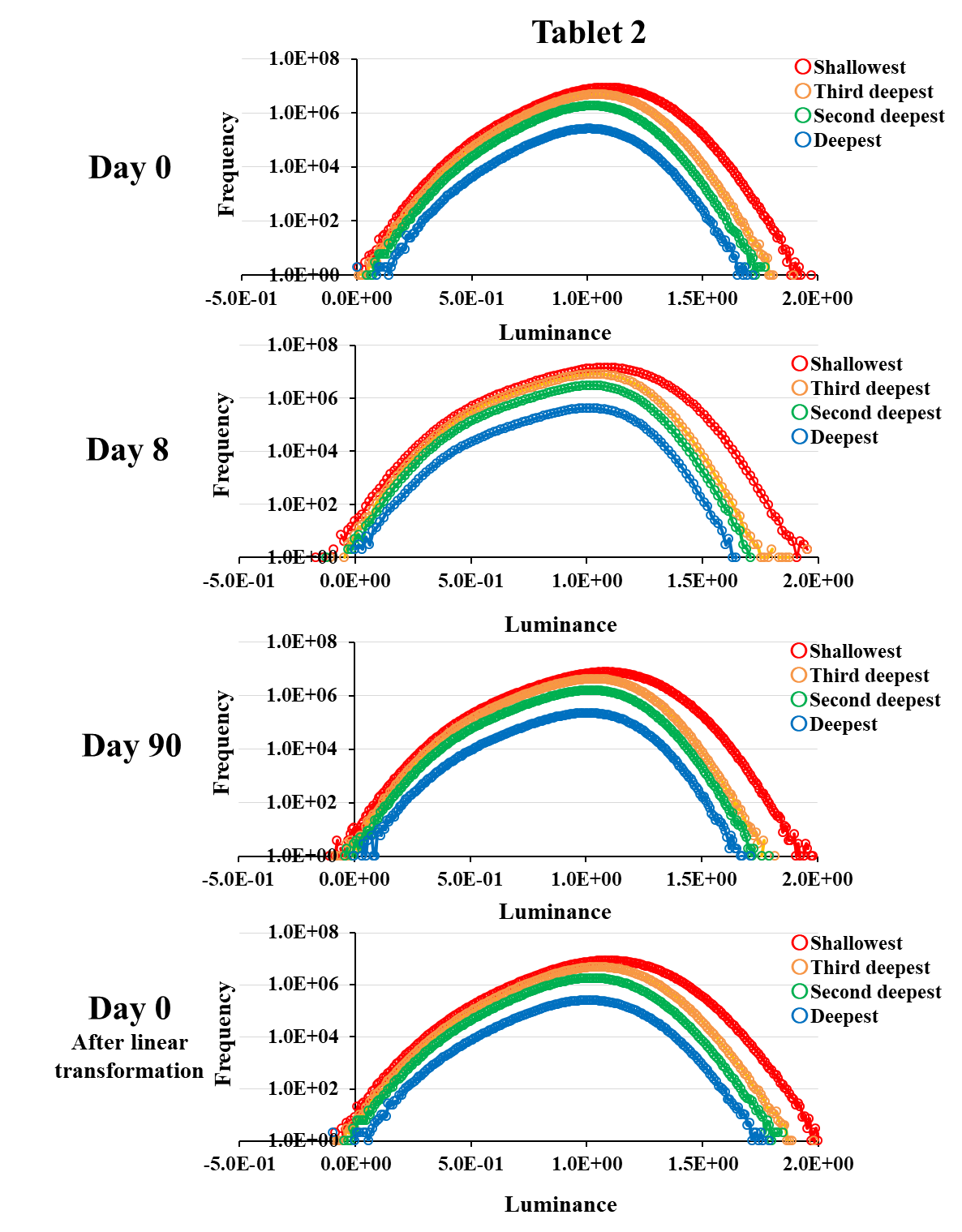


(c) Cumulative frequency distributions of the histograms presented in (b).


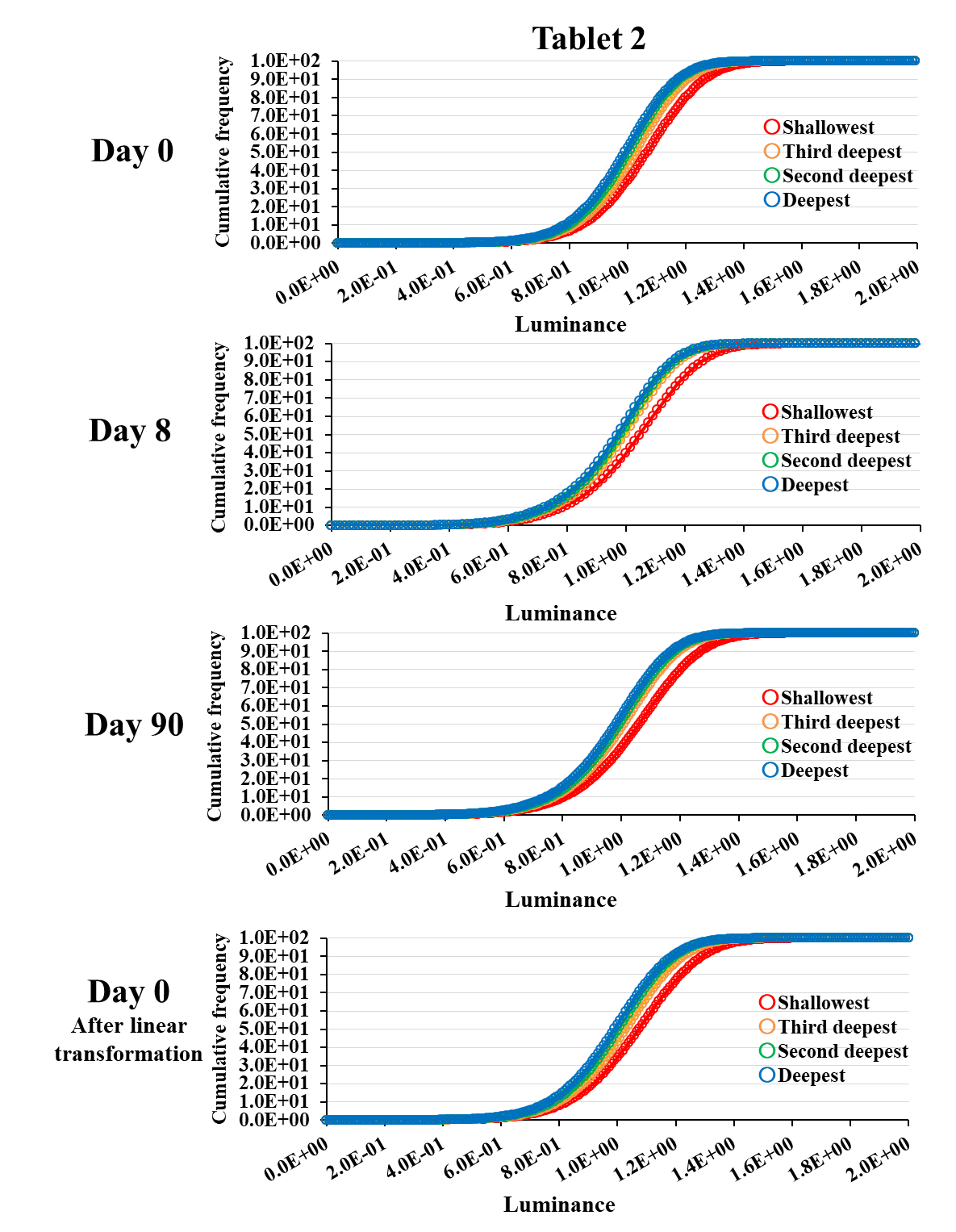

Supplement: Supplementary file 1 — Supplementary Figures. [file 41598_2024_56949_MOESM1_ESM.docx]
